# Prevalence of *Escherichia Coli* and Its Antimicrobial Susceptibility Profiles among Patients with UTI at Mulago Hospital, Kampala, Uganda

**DOI:** 10.1155/2020/8042540

**Published:** 2020-02-01

**Authors:** Isaac Odongo, Ronald Ssemambo, Joseph M. Kungu

**Affiliations:** College of Veterinary Medicine, Animal Resources and Biosecurity Makerere University, P. O. Box 7062, Kampala, Uganda

## Abstract

**Background:**

Urinary tract infections (UTIs) remain the most common infections diagnosed in in- and outpatients as well as hospitalized patients. Current knowledge on antimicrobial susceptibility pattern for uropathogens is essential to effectively manage UTIs. This study aimed at determining the prevalence of *E. coli* and its antimicrobial susceptibility profiles among patients presenting with signs and symptoms of UTI in Mulago Hospital in Uganda.

**Methods:**

Midstream urine samples were collected from 100 patients presenting with signs and symptoms of UTI at the outpatient department of Mulago Hospital. The samples were cultured, and isolates of *E. coli* and its antimicrobial susceptibility profiles among patients presenting with signs and symptoms of UTI in Mulago Hospital in Uganda.

**Results:**

Out of 100 patients studied, *E. coli* and its antimicrobial susceptibility profiles among patients presenting with signs and symptoms of UTI in Mulago Hospital in Uganda. *Escherichia coli* isolates were highly susceptible to cefotaxime/clavulanic acid (100%) and nitrofurantoin (70%) but showed high resistance to cefuroxime (100%), ceftazidime (100%), nalidixic acid (90%), and ciprofloxacin (90%).

**Conclusion:**

*Escherichia coli*, the predominant uropathogen, showed significant multidrug resistance to antibiotics commonly prescribed for the management of UTIs. These findings should form a basis for preliminary decision making on the appropriate line of treatment for UTIs.*Escherichia coli* isolates were highly susceptible to cefotaxime/clavulanic acid (100%) and nitrofurantoin (70%) but showed high resistance to cefuroxime (100%), ceftazidime (100%), nalidixic acid (90%), and ciprofloxacin (90%).

## 1. Background

Urinary tract infections (UTIs) are among the most common bacterial infections encountered in primary health care, and it is among the most common infections with an increasing resistance to antimicrobial agents [1]. These infections have also become the most common hospital-acquired infections, accounting for up to 35% of nosocomial infections and second cause of bacteremia in hospitalized patients. This ailment affects patients in all age groups and sexes, with females accounting for 87.5% of the cases compared with males (71.3%) [2–4]. This is associated with the short urethral tube of women and close proximity of the anus to the urethral opening, allowing easier access of the bacteria to the urethra. It is estimated that half of all women will have recurrent episodes of acute cystitis during adult life [5].

The enterobacterales order is the most common etiological agent of urinary tract infections because they have several factors associated with their attachment to the uroepithelium such as possession of adhesins [6]. *Escherichia coli* has been documented to be the most common pathogen associated with urinary tract infections in many countries causing both community- and hospital-acquired UTI [7, 8]. Other pathogens such as *Enterococcus* sp., *Staphylococcus saprophyticus* (“honeymoon cystitis”), *Klebsiella* sp., *Enterobacter* sp., *Citrobacter* sp., and *Proteus-Morganella-Providencia* sp. may also occur [9].

The emergence and spread of antibiotic-resistant pathogens is a major public health threat. Resistant pathogens especially enterobacteriaceae can withstand lethal doses of antibiotics with various chemical structures and mechanisms of action [10]. Enterobacteriaceae such as *E. coli* exhibit important mechanisms to avoid lethal doses of drugs such as aminoglycoside degrading enzyme, target alteration, decreased uptake, beta lactamase enzyme degradation, and overexpression of efflux proteins. The rate of drug discovery and its development in the 21st century cannot match with the continuous and detrimental change in antibiotic resistance trends [11]. Since the introduction of fluoroquinolones in 1960s in an attempt to optimise nalidixic acid, there has been no broad-spectrum antibiotics discovered and developed. Pathogens associated with UTI are increasingly changing their features particularly due to self-medication, overuse, and misuse of drugs [12]. With the rampant antimicrobial misuse, there is a rise in the newer and more resistant strains of the etiological agents of UTI. Bacterial infections resistant to antibiotics can limit effective treatment rendering bacterial infections difficult to treat including UTI. In low-income countries, there is a reduced access to health care and high cost of second line drugs which limits the use of newer broad-spectrum drugs [2].

The resistance patterns of community-acquired UTI have not been studied exclusively, yet knowledge regarding common uropathogens and their susceptibility patterns to drugs is key in improving prescription decisions [12]. Treatment of patients at Mulago hospital is usually carried out empirically and regular laboratory culture and sensitivity tests of urine from UTI patients are limited [13].

## 2. Methods

### 2.1. Study Design

A cross-sectional study was conducted from January to March 2018, to determine the prevalence and antimicrobial susceptibility profiles of *E. coli* among patients presenting with UTI at the outpatient's department of Mulago National Referral Hospital, Kampala, Uganda.

All patients with signs and symptoms of UTI who voluntarily consented were recruited in the study. Participants who qualified for the study after signing the consent forms provided midstream urine samples, and information about their age and sex was gathered. The urine samples were cultured to determine the presence of *E. coli* organisms. Positive samples for *E. coli* were further subjected to antimicrobial susceptibility testing to evaluate their antimicrobial-susceptible profiles.

### 2.2. Sample Size Determination

The sample size was determined using a formula by Thrusfield as follows [14]:

(1) $$\begin{array}{c} n=\frac{z^{2}p\left(1-p\right)}{i^{2}}, \end{array}$$

where *n* is the calculated sample size, *z* is the desired level of confidence (1.96), *i* is the standard sampling error (10%), and *p* is the estimated prevalence 50% [15]. Although a minimum sample size required was 96, up to 100 patients from Mulago Hospital who met the inclusion criteria were recruited in the study to increase precision.

### 2.3. Collection and Analysis of Urine Samples

A total of 100 midstream urine samples were collected into a sterile urine container on the same day of enrollment. The samples were sent to the laboratory for analysis, and most of the samples were analyzed within one hour after collection.

10 *μ*l of well-mixed urine samples were inoculated on MacConkey agar (Oxoid limited, United Kingdom) using a sterile loop following standard culture procedures. The plates were incubated at 37°C for 24 hours. Morphological colony identification and biochemical tests were used to confirm the *E. coli* organisms. Disc diffusion method was used to determine the antibiotic susceptibility of *E. coli. Escherichia coli* isolates were suspended in peptone water and incubated at 37°C until turbid and turbidity adjusted to a standard uniform concentration of 0.5 McFarland solutions. The isolates were then inoculated on Mueller Hinton agar (Oxoid, United Kingdom). The antibiotic discs containing precise concentration of the antibiotics were individually placed 1 cm from the wall and from each other. The plates were then incubated at 37°C for 24 hours. The diameter zones of clearance were measured in millimetres and interpreted according to the Clinical Laboratory Standard Institute (CLSI) protocol [16]. For quality control, *E. coli* ATCC 25922 provided in the laboratory was used as a control strain.

### 2.4. Data Analysis

The data from the study were entered and cleaned in MS Excel and analyzed using SPSS software version 20. The Chi-squared test was used to perfoFrm descriptive statistics at 95% level of confidence (Table 1).

## 3. Results

### 3.1. Prevalence of *Escherichia coli* and Other Bacteria among UTI Patients

Out of the 100 samples collected, *E. coli* was the most dominant pathogen at 10%. There were other isolates identified in the urine samples which included; *C. freundi* (2%), *K. pneumoniae* (2%), *S. aureus* (1%), *P. aeruginosa* (1%), and *Streptococcus* sp. (1%). More females (52%) participated in the study compared with the males (48%). The prevalence of *E. coli* was higher among the females (6%) compared with the males (4%). Furthermore, the prevalence was also higher in the age group of 0–17 years (4%) compared with other groups as described in Table 2.

Cross tabulations of demographic variables (age and sex) with prevalence of *E. coli*, using Chi-squared test was performed at 95% level of confidence. There were no significant differences between the variables and *E. coli* prevalence.

### 3.2. Antimicrobial Susceptibility of *E. coli* Isolates to the Common Antibiotics

All the *E. coli* isolates were sensitive cefotaxime/clavulanic acid (100%). Most of the isolates were sensitive to nitrofurantoin (70%), and few showed sensitivity to nalidixic acid and ciprofloxacin (10%). However, all the isolates showed significantly high sensitivity to cefuroxime and ceftazidime (100%). The results of the pattern of antibiotic susceptibility are shown in Table 3.

## 4. Discussion

Up to date information on prevalence and antibiotic susceptibility pattern of pathogenic bacteria is essential in therapeutic management of UTIs. This study established that prevalence of *E. coli* among UTI patients was fairly low (10%) compared with other related studies. In a previous study conducted in patients attending hospitals in Bushenyi district western Uganda by Odoki and others, a significantly high prevalence of *E. coli* 41.9% was reported [13]. Likewise, previous related studies in Mulago Hospital showed a higher prevalence of 57.5 and 50%, respectively [15, 17]. The prevalence of this study was low compared with the previous possibly due to population variation and significant differences in the sample sizes. It is also possible that with time, tremendous improvements in management of UTIs and community hygiene could have contributed to reduction of the prevalence of *E. coli* organisms [15].

The prevalence was clearly high in females (11.5%) compared with the males (8.3%) though it was not statistically significant. A number of previous studies also documented the prevalence of *E. coli* in UTI patients to be high in females compared with males [2, 6, 13, 18, 19]. This could be due to the close proximity of anus to the warm urethral tube. Furthermore, the urethral tube of the females is short, and this shortens the distance moved by the organism to the bladder. These predisposing factors of UTI are accelerated by limited resources, poor hygiene, and low socioeconomic status [2]. Alteration in the vaginal microflora significantly play an important role in encouraging colonization of the vagina with coliforms, and this can be associated with UTI [20].

The prevalence was revealed to be high in the age group of ≤17 years, 11.4%, compared with other age groups though it was not statistically significant. The findings are similar to the findings reported in Nigeria [21]. The juveniles have low immunity against infections and could never have been exposed to the infections before, and this might have highly exposed them to the infections. Furthermore, young children in most cases in our setting are misdiagnosed since symptoms of UTI in infants resemble the symptoms of other infections such as fever, vomiting, and refusing to eat. Those in adolescence age are involved in increased sexual activity which predisposes them to UTI [13, 21].

*Escherichia coli* isolated showed a high sensitivity of 100% to cefotaxime/clavulanic acid. This could be due to the fact the drug combinations are not easily resisted by the organism. The clavulanic acid targets the enzyme beta lactamase that is responsible for resistance against beta lactam antibiotics, and this helps the drug to overcome resistance; combination of cephalosporins and beta lactamase inhibitor are among the first new drug formulations that may possess clinically relevant broad-spectrum antibacterial activity. Furthermore, it has been rarely used in the treatment of urinary tract infections and other infections and therefore present organisms present low resistance to it since they have not been frequently exposed to it [1, 6, 22].

The results from the study also showed that 70% of the isolates were susceptible to nitrofurantoin. An earlier study in the same study setting involving nonpregnant women had shown high sensitivity of *E. coli* to nitrofurantoin at 100% sensitivity rates [17]. Likewise, another study reported a similar susceptibility of 78% [15]. This shows a decline in the sensitivity rates of *E. coli* to this antibiotic. The increasing resistance could be due to increased overuse and misuse of antibiotics. The persistor cells (defined as metabolically inactive cells that neither grow or die when exposed to bactericidal concentrations of the antibiotics) presents another important challenge as these cells tend to be associated with treatment failure, recurrence, and chronic infections as they continue to replicate after the antibiotic therapy is discontinued [23]. It could also possibly due to the cheap costs and ready availability of this drug since previous Uganda Clinical Guidelines 2016 recommended it in the empirical treatment of urinary tract infections [13].

*Escherichia coli* isolates were highly resistant to cefuroxime and ceftazidime, and 100% was so alarming in this study. The resistance in a previous study in Mulago Hospital was significantly lower [15]. Resistance to nalidixic acid and ciprofloxacin was also alarming (90%), and this was similar to a related study in Mulago which reported 89.9% resistance of *E. coli* [17]. Ciprofloxacin has always been used in the empirical treatment of urinary tract infections. This resistance could be due to previously increased use of these drugs since the previous Uganda Clinical Guidelines 2010 recommended its use in the empirical treatment of UTI, and also these drugs are relatively cheaper and readily available. This could have rendered them easily accessible to the patients, increasing their misuse and overuse, leading to resistance.

## 5. Conclusion

*Escherichia coli*, the predominant organism, observed in the UTI patients was low. There was a significantly high resistance to cefuroxime, ceftazidime, nalidixic acid, and ciprofloxacin to *E. coli* isolated. Continuous use of these drugs might most likely be associated with treatment failure and serious antimicrobial resistance. There was acceptably high sensitivity to cefotaxime/clavulanic acid and nitrofurantoin. This study recommends that urine culture and sensitivity should be done when UTI is strongly suspected to guide clinicians and physicians in treatment decisions. The study recommends that either nitrofurantoin and cefotaxime-clavulanic acid or their combination be used in the empirical treatment of UTI in the Ugandan settings. There should be continuous periodic antibiotic resistance monitoring to curb resistance emergence.

## Figures and Tables

**Table 1:** Prevalence of *E. coli* and other bacteria among UTI patients in Mulago Hospital.

Isolate	Number of isolates	Percent
*E. coli*	10	10
*C. freundi*	2	2
*K. pneumoniae*	2	2
*Streptococcus spp*	1	1
*P. aeruginosa*	1	1
*S. aur*eus	1	1

**Table 2:** Cross tabulation of demographic variables with prevalence of *E. coli*.

Variable	Category	*E. coli* (*+*)	*E. coli* (*−*)	Prevalence	*X* ^2^	*P* value
Sex	Male	4	44	8.3	0.285	0.594
	Female	6	46	11.5		
Age groups in years	0–17	4	31	11.4	0.635	0.88
	18–30	2	28	6.7		
	31–50	2	18	10		
	>51	2	13	13.3		

**Table 3:** Antibiotic susceptibility findings of the *E. coli* isolated from urine samples of UTI patients in Mulago Hospital.

Antibiotic	Concentration of antibiotic in the disc (*μ*g)	Resistant	Susceptible
Ciprofloxacin	5	9 (90.0%)	1 (10.0%)
Ceftazidime	30	10 (100%)	0 (0.0%)
Cefotaxime/clavulanic acid	30/10	0 (0.0%)	10 (100%)
Nalidixic acid	30	9 (90.0%)	1 (10.0%)
Nitrofurantoin	300	3 (30.0%)	7 (70.0%)
Cefuroxime	30	10 (100%)	0 (0.0%)

## Data Availability

The data used to support the findings of this study are included within the article.

## Abbreviations

CLSI: Clinical Laboratory Standard Institute Protocol (CLSI)
UTI: Urinary Tract Infections.
